# Surgical treatment of appendiceal mucormycosis in an immunocompromised patient: a case report

**DOI:** 10.1186/s40792-024-01958-y

**Published:** 2024-06-25

**Authors:** Yukiya Orihara, Shingo Kurahashi, Katsuhiko Kamei, Kazuhiro Hiramatsu

**Affiliations:** 1 Department of General Surgery, Toyohashi Municipal Hospital, 50 Hachikennishi, Aotake-Cho, Toyohashi, Aichi Japan; 2 Department of Hematology, Toyohashi Municipal Hospital, 50 Hachikennishi, Aotake-Cho, Toyohashi, Aichi Japan; 3 Division of Infection Control and Prevention, Medical Mycology Research Center, Chiba University, 1-8-1 Inohana, Chuo-Ku, Chiba, Chiba Japan

**Keywords:** Mucormycosis, Rhizopus microspores, Gastrointestinal mucormycosis

## Abstract

**Background:**

Gastrointestinal mucormycosis is a rapidly progressing and often fatal disease, predominantly affecting immunocompromised patients. Surgical intervention, in addition to antifungal therapy, is essential. Herein, we describe the successful management of appendiceal mucormycosis in a patient with acute promyelocytic leukemia through rapid surgical intervention and antifungal therapy.

**Case presentation:**

A 29-year-old woman underwent autologous peripheral blood stem cell transplantation for acute promyelocytic leukemia (APL). Subsequently, her condition relapsed, and remission induction therapy was initiated. During the immunosuppressive period, she developed a fever and severe abdominal pain. Computed tomography revealed severe edema of the ileum, cecum, and ascending colon. Despite receiving multiple antibiotics, antivirals, and antifungals, her condition showed no improvement. Consequently, she underwent exploratory laparotomy, with no bowel perforation noted, revealing severe inflammation in the ileum, cecum, and ascending colon, as well as appendiceal necrosis. Appendectomy was performed, and histopathological analysis revealed hyphae in the vessels and layers of the appendiceal wall, suggestive of mucormycosis. The patient was diagnosed with appendiceal mucormycosis, and liposomal amphotericin B was administered. Subsequent monitoring showed no recurrence of mucormycosis. Genetic analysis of the resected tissue revealed *Rhizopus microspores* as the causative agent.

**Conclusions:**

Rapid surgical intervention and antifungal drug administration proved successful in managing appendiceal mucormycosis in a patient with APL. Early recognition and aggressive surgical intervention are imperative to improve outcomes in such patients.

## Background

Mucormycosis, which is caused by fungi of the Mucorales order [1], is a rare yet rapidly progressing condition, necessitating immediate intervention. The all-cause mortality rates of mucormycosis range from 40 to 80% [1]. The underlying diseases include hematologic malignancies, trauma, hematopoietic stem cell transplantation, and diabetes mellitus. Of these, hematological malignancies are the most common [2]. Mucormycosis-related diseases manifest in various forms, including rhinocerebral, pulmonary, cutaneous, gastrointestinal, disseminated, or others [3]. The frequency of gastrointestinal mucormycosis is reported to be 5–15% [4]. While the stomach is the most common site of gastrointestinal mucormycosis, followed by the colon and the ileum, appendiceal mucormycosis is an extremely rare condition. To the best of our knowledge, there are three detailed reports of appendiceal mucormycosis in adults [5–7]. Two of the three cases have hematologic malignancies, and all three are on chemotherapy.

Surgical intervention is recommended as early as possible for mucormycosis, especially for the skin, rhinocerebral, and pulmonary forms [1, 8, 9]. Surgical intervention is associated with increased survival [2]. However, reports of surgical interventions for gastrointestinal mucormycosis are limited.

In this report, we describe a case of appendiceal mucormycosis in a patient with acute promyelocytic leukemia (APL) who had good prognosis after surgical intervention and antifungal therapy with liposomal amphotericin B (L-AMB).

## Case presentation

**Fig. 1:**
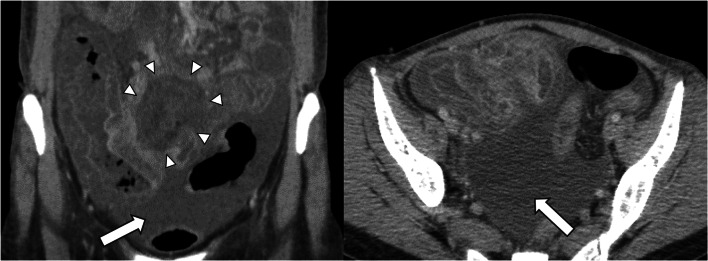
Abdominal contrast-enhanced computed tomography (CT). CT shows marked wall thickening from the ileum to the ascending colon, with massive ascites (arrow) and a low-density area suspicious for an abscess (arrowhead). The appendix was not contrasted and could not be recognized

**Fig. 2:**
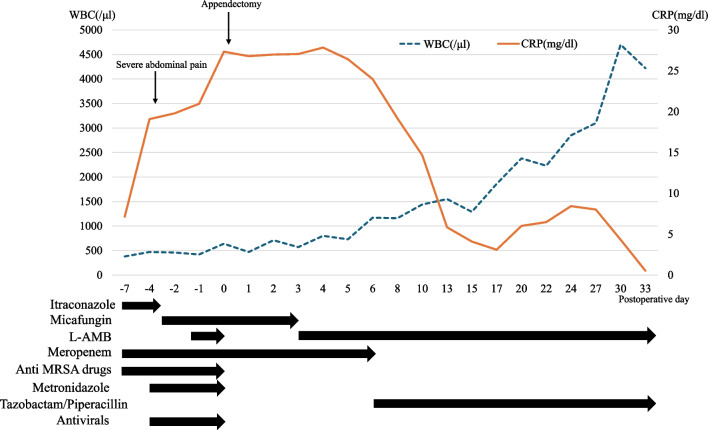
Perioperative course of the treatment and inflammatory response. The inflammatory response improved with surgery and L-AMB administration

A 29-year-old woman who was diagnosed with APL achieved complete response (CR) with remission induction therapy, including all-trans retinoic acid, followed by consolidation therapy. Despite these treatments, she experienced hematological relapse, necessitating remission induction therapy including arsenic trioxide (ATO), resulting in another CR, and subsequently underwent autologous peripheral blood stem cell transplantation. However, the patient showed hematological relapse and was treated with induction remission therapy with ATO and idarubicin. She experienced prolonged myelosuppression and was severely immunosuppressed. Subsequently, she developed fever and severe abdominal pain. Computed tomography (CT) revealed ascites and severe edema of the ileum, cecum, and ascending colon (Fig. 1). The appendix was not contrasted and could not be recognized. Meropenem, vancomycin, and metronidazole were administered as antibacterial agents, acyclovir and ganciclovir as antivirals, and micafungin and L-AMB as antifungals (Fig. 2). Laboratory findings indicated severe pancytopenia, neutrophil count of 109/µL, hemoglobin level of 7.7 g/dL, and platelet count of 15,000/µL, with a highly elevated inflammatory response with a C-reactive protein (CRP) level of 27.34 mg/dL. Therefore, she was administered red blood cell and platelet transfusions, granulocyte-colony stimulating factor, and intravenous immunoglobulin.

**Fig. 3:**
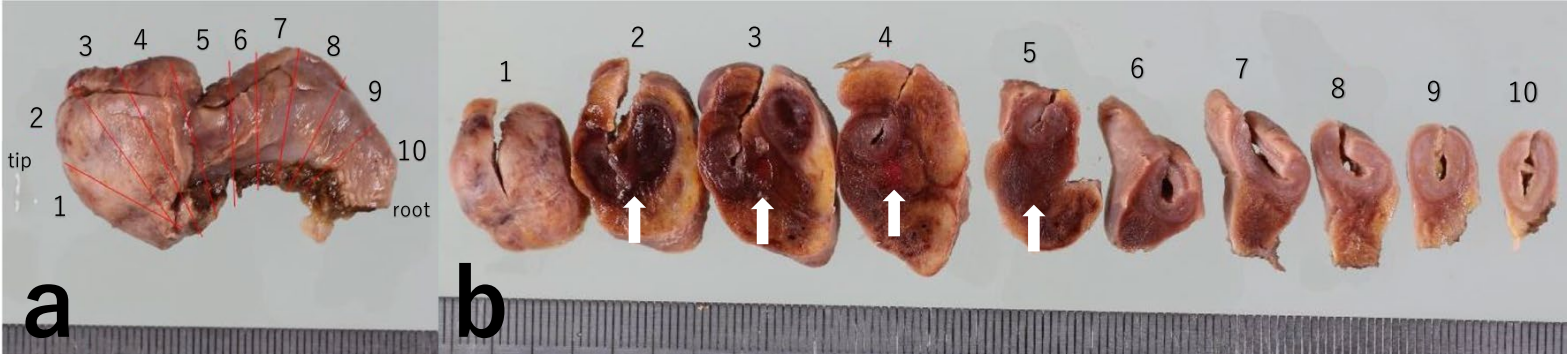
Macroscopic findings. **a** The appendix is enlarged, particularly from the middle to the tip. **b** Resected appendiceal specimen. Wall thickening and hemorrhaging can be seen in the appendix and surrounding fatty tissue (arrow). The appendix was found to be infected and necrotic from the root to the tip

**Fig. 4:**
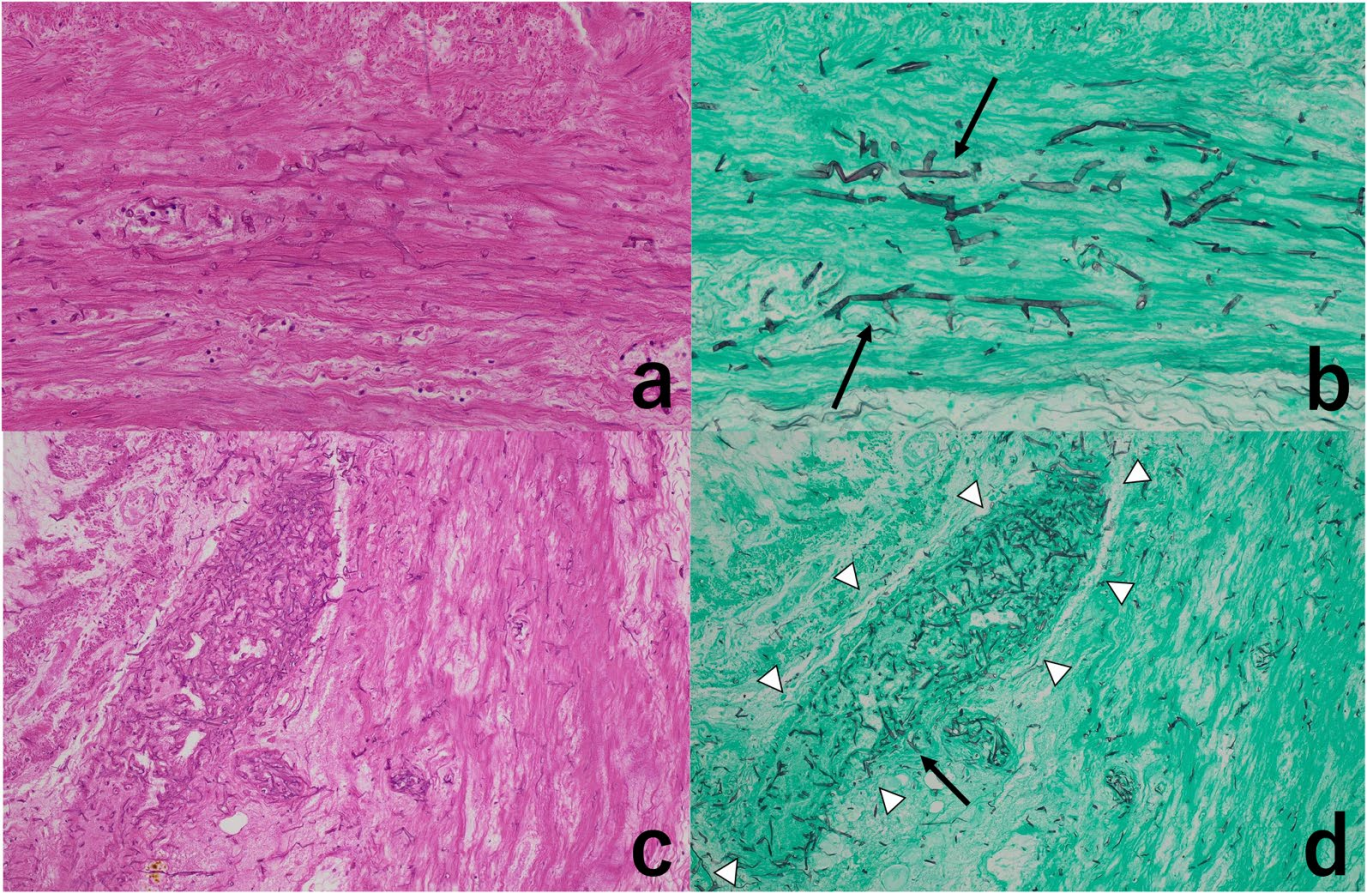
Microscopic findings of the appendix. **a** Inflammatory cell infiltration is almost absent within the appendiceal wall tissue (hematoxylin and eosin staining ×200). **b** Hyphae are present in the submucosa and branch at an angle of about 90° (arrow) (Grocott’s staining ×200). **c** There is little inflammatory cell infiltration within the small arteries or surrounding tissue (hematoxylin and eosin staining ×100). **d** Mucorales infiltrates from the vessel wall into the vessel lumen (arrow). The small arteries are filled with hyphae (arrowhead) (Grocott’s staining ×100)

After four days of conservative treatment with no improvement, exploratory laparotomy was performed, accessing the lower abdomen through a midline incision. Because of sepsis and the need for rapid and reliable source control, laparotomy was chosen instead of laparoscopy. Although there was no evidence of bowel perforation, severe inflammation of the ileum, cecum and ascending colon and necrosis of the appendix were observed. As the appendix was the main site of inflammation, appendectomy was performed, with an operative time of 115 min and minimal blood loss. The resected specimen was all necrotic, with marked wall thickening (Fig. 3). Microscopically, the hyphae were in the submucosa, showing branching at about 90°, suggesting mucor. The small arteries were filled with hyphae and showed hyphae embolization (Fig. 4). Blood tests showed negative results for β-d glucan and Aspergillus GM antigen; thus, appendiceal mucormycosis was strongly suspected. Genetic analysis of the resected tissue using panfungal primers to target the fungal ribosomal RNA genes revealed that *Rhizopus microsporus* was the causative fungus. Until postoperative day 2, she was treated with antimicrobials because of suspected bacterial appendicitis. L-AMB therapy was started on postoperative day 3 after histopathological examination results were available. Postoperatively, the neutrophil count recovered gradually, and the CRP level showed a decreasing trend (Fig. 2). No postoperative complication, such as surgical site infection, was noted. L-AMB therapy was terminated on day 56. Prior to the end of treatment, colonoscopic biopsy was performed for confirmation of no residual lesion. The patient underwent allogeneic hematopoietic stem cell transplantation for APL, with no evidence of mucormycosis recurrence.

## Discussion

Despite the high perioperative risk, aggressive surgical intervention for gastrointestinal mucormycosis can help in improving prognosis. When immunocompromised patients who have undergone organ or stem cell transplantation have hematologic malignancies or diabetes mellitus or are on steroids present with an acute abdomen, we must consider the involvement of fungi, especially Mucorales, as well as bacterial causes. However, early diagnosis of gastrointestinal mucormycosis is extremely difficult, as its symptoms and imaging findings are nonspecific. In the past, the disease was often detected during autopsy; however, in recent years, it has been detected earlier more frequently [10]. Nevertheless, gastrointestinal mucormycosis is rarely diagnosed preoperatively and is most often discovered postoperatively, as in our case.

Patients prone to mucormycosis generally have a higher perioperative risk. The patient in our study also had bone marrow suppression due to a hematologic malignancy and was at risk of perioperative infection due to neutropenia and postoperative bleeding due to thrombocytopenia. The mortality rate of surgical intervention is approximately five times higher in patients with hematologic malignancies than in the general surgical population, necessitating careful consideration to determine whether surgical intervention should be performed [11]. However, surgical intervention, in addition to antifungal agents, can improve the prognosis of patients with mucormycosis [12]. Mucormycosis causes vascular invasion, thrombosis, and tissue necrosis, preventing antifungal penetration at the infection site [8, 12]. In some cases, there may be no response to antifungal drugs despite susceptibility to these agents. In such cases, prompt consideration should be given to early surgical intervention aimed at debriding the Mucor-infected site. Although it is desirable to resect as many lesions as possible to increase the efficacy of antifungals, it is difficult to determine the extent of resection. Rapid intraoperative pathology has been reported to be helpful in determining the resection margins [13] and may be useful for mucormycosis of the colon and small intestine. In our case, Mucorales was present at root of the appendix. Although there was a high possibility that Mucorales remained in the cecum, postoperative administration of antifungal agents led to the cure of the disease. Possible reasons for this are that the site of hyphae embolization due to vascular invasion was localized to the appendix and the residual lesions were well penetrated by antifungal agents. This is suggested by the absence of a contrast effect in the appendix on preoperative CT but the presence of a contrast effect in the ileum, cecum, and ascending colon.

Delayed administration of antifungal agents for mucormycosis can significantly increase mortality [14]. In immunocompromised patients presenting with an acute abdomen, antibiotics and antifungals, including L-AMB, should be administered first, with mucormycosis in consideration. If a patient does not respond to drug treatment and there is no improvement in the condition, surgical intervention should be administered promptly. If a diagnosis of mucormycosis is made, surgery should be performed immediately; however, mucormycosis is difficult to diagnose. Moreover, the criteria for considering surgical intervention in cases of undiagnosed but suspected mucormycosis have not been established. Considering the speed of mucormycosis progression, evaluation and treatment must be performed promptly if mucormycosis is suspected.

Although many studies have reported on the efficacy of surgical intervention for the rhinocerebral and pulmonary forms of mucormycosis [15–17], reports on surgical intervention for gastrointestinal mucormycosis are limited. However, in the present case, emergency surgery was performed within approximately 4 days of the onset of abdominal pain, which allowed for the control of inflammation, identification of the fungus, and selection of antifungals accordingly. These results suggest the effectiveness of surgical intervention in the treatment of gastrointestinal mucormycosis.

## Conclusions

Herein, we report a case of successful surgical intervention for appendiceal mucormycosis in a patient with myelosuppression secondary to acute promyelocytic leukemia. The acute abdomen in immunocompromised patients may be caused by Mucorales, warranting consideration of aggressive surgical interventions, such as debridement, for such patients.

## Data Availability

Not applicable.

## Abbreviations

APL: Acute promyelocytic leukemia
L-AMB: Liposomal amphotericin B
CR: Complete response
ATO: Arsenic trioxide
CT: Computed tomography
CRP: C-reactive protein
